# MELOGEN: an EST database for melon functional genomics

**DOI:** 10.1186/1471-2164-8-306

**Published:** 2007-09-03

**Authors:** Daniel Gonzalez-Ibeas, José Blanca, Cristina Roig, Mireia González-To, Belén Picó, Verónica Truniger, Pedro Gómez, Wim Deleu, Ana Caño-Delgado, Pere Arús, Fernando Nuez, Jordi Garcia-Mas, Pere Puigdomènech, Miguel A Aranda

**Affiliations:** 1 Departamento de Biología del Estrés y Patología Vegetal, Centro de Edafología y Biología Aplicada del Segura (CEBAS)- CSIC, Apdo. correos 164, 30100 Espinardo (Murcia), Spain; 2 Departamento de Biotecnología, Instituto de Conservación y Mejora de la Agrodiversidad Valenciana (COMAV-UPV), Camino de Vera s/n, 46022 Valencia, Spain; 3 Departament de Genètica Vegetal, Centre de Recerca en Agrigenòmica CSIC-IRTA, Carretera de Cabrils Km2, 08348 Cabrils (Barcelona), Spain; 4 Departament de Genètica Molecular, Centre de Recerca en Agrigenòmica CSIC-IRTA, Jordi Girona 18-26, 08034 Barcelona, Spain

## Abstract

**Background:**

Melon (*Cucumis melo *L.) is one of the most important fleshy fruits for fresh consumption. Despite this, few genomic resources exist for this species. To facilitate the discovery of genes involved in essential traits, such as fruit development, fruit maturation and disease resistance, and to speed up the process of breeding new and better adapted melon varieties, we have produced a large collection of expressed sequence tags (ESTs) from eight normalized cDNA libraries from different tissues in different physiological conditions.

**Results:**

We determined over 30,000 ESTs that were clustered into 16,637 non-redundant sequences or unigenes, comprising 6,023 tentative consensus sequences (contigs) and 10,614 unclustered sequences (singletons). Many potential molecular markers were identified in the melon dataset: 1,052 potential simple sequence repeats (SSRs) and 356 single nucleotide polymorphisms (SNPs) were found. Sixty-nine percent of the melon unigenes showed a significant similarity with proteins in databases. Functional classification of the unigenes was carried out following the Gene Ontology scheme. In total, 9,402 unigenes were mapped to one or more ontology. Remarkably, the distributions of melon and Arabidopsis unigenes followed similar tendencies, suggesting that the melon dataset is representative of the whole melon transcriptome. Bioinformatic analyses primarily focused on potential precursors of melon micro RNAs (miRNAs) in the melon dataset, but many other genes potentially controlling disease resistance and fruit quality traits were also identified. Patterns of transcript accumulation were characterised by Real-Time-qPCR for 20 of these genes.

**Conclusion:**

The collection of ESTs characterised here represents a substantial increase on the genetic information available for melon. A database (MELOGEN) which contains all EST sequences, contig images and several tools for analysis and data mining has been created. This set of sequences constitutes also the basis for an oligo-based microarray for melon that is being used in experiments to further analyse the melon transcriptome.

## Background

Melon (*Cucumis melo *L.) is an important horticultural crop grown in temperate, subtropical and tropical regions worldwide. Melon is among the most important fleshy fruits for fresh consumption, its total production in 2004 exceeding 874 million metric tons, of which 72.5% are produced in Asia, 11.7% in Europe, 8.4% in America and 6.1% in Africa, being a significant component of fresh fruit traded internationally [1]. Melon belongs to the *Cucurbitaceae *family, which comprises up to 750 different species distributed in 90 genera. Species in this family include watermelon, cucumber, squash and marrow, all of them cultivated essentially because of their fruits, but this family also includes species of interest for other reasons, as, for example, their contents in potentially therapeutic compounds (e.g. *Momordica charantia*) [2]. Melon is a diploid species, with a basic number of chromosomes x = 12 (2x = 2n = 24) and an estimated genome size of 450 to 500 Mb [3], similar in size to the rice genome (419 Mb) [4,5] and about three times the size of the Arabidopsis genome (125 Mb) [6]. Melon has been classified into two subspecies, *C. melo *ssp. *agrestis *and *C. melo *ssp. *melo *with India and Africa being their centres of origin, respectively [7,8].

Melon has a great potential for becoming a model for understanding important traits in fruiting crops. Melon fruits have wide morphological, physiological and biochemical diversity [7,9] which can be exploited to dissect biological processes of great technological importance, among them flavour development and textural changes that occur during fruit ripening. The contemporary melon cultivars can be divided into two groups, climacteric and nonclimacteric, according to their ripening patterns [10]. Climacteric fruits are characterized by rapid and profound changes during ripening associated to increased levels of respiration and release of ethylene, whereas the nonclimacteric varieties do not produce ethylene and have long shelf-life. Analyses of climacteric and nonclimacteric melons have illustrated the process of aroma formation [11-14] and the temporal sequence of cell wall disassembly [15-17]. Melon can be also a very useful experimental system to analyse other aspects of fundamental plant biology. For example, melon and other cucurbits have been used to analyse the development of the plant vasculature and the transportation of macromolecules through it [18-20], and different interactions between melon and pests and pathogens have been characterised with varying depths [21-27].

Important genetic tools have been described for melon, as for example linkage genetic maps [28,29] and the development of a genomic library of near isogenic lines (NILs) from an exotic accession [30]; also, biotechnology is feasible in melon [31-33]. However, the great majority of genes involved in the aforementioned traits are yet to be identified in melon. Partial sequencing of cDNA inserts of expressed sequence tags (ESTs) have been used as an effective method for gene discovery. By sequencing clones derived from RNA from different sources, and/or by normalizing cDNA libraries, the total set of genes sampled can be maximized. Bioinformatic analysis, annotation and clustering of sequences could yield databases which mining can be used to select candidate genes implicated in traits of interest. EST collections can also serve to construct microarrays useful for identifying sets of plant genes expressed during different developmental stages and/or responding to environmental stimuli [34,35]. In addition, EST collections are good sources of simple sequence repeats (SSRs) and single-nucleotide polymorphisms (SNPs) that can be used for creating saturated genetic maps [36,37]. Thus, EST collections have been generated for many plant species, being the most comprehensive those of Arabidopsis[6] and rice [38]. Fruit crops have been less extensively surveyed, but important collections are publicly available for several species, including tomato [39], apple [40], grape [41] and citrus [42].

Despite the importance of the family *Cucurbitaceae*, relatively little EST information is currently available: only 16,039 nucleotide sequences have been annotated from the whole *Cucurbitaceae *family in the publicly accessible GenBank database as of November 2006; out of these, 12,180 correspond to the *Cucumis *genus and 6,061 to melon. These numbers are in sharp contrast with the data available for families composed of other important food crops like *Solanaceae *(1,020,102 sequences), *Fabaceae *(1,466,518 sequences), *Brassicaceae *(1,010,148 sequences excluding Arabidopsis), *Vitaceae *(449,478) and *Rosaceae *(390,066 sequences). Here we describe a public EST sequencing project in melon. We report the determination and analysis of 30,675 high-quality melon ESTs, sequenced from eight normalized cDNA libraries corresponding to different tissues in different physiological conditions. We have classified the sequences into functional categories and described SSRs and SNPs of potential use in genetic maps and marker-assisted breeding programs. A database which contains all EST sequences, contig images and several tools for analysis and data mining has been created. In addition, we have analyzed the EST melon dataset to identify candidate genes potentially coding microRNAs or involved in fruit maturation processes and pathogen defence. The pattern of transcript accumulation in different physiological conditions has been characterised by Real-Time-qPCR for 20 of these candidate genes.

## Results

### EST Sequencing and Clustering

Eight cDNA libraries were constructed using material from "Piel de Sapo" Spanish cultivars, the C-35 cantaloupe line (both belonging to *Cucumis melo *L. ssp. *melo*) and the accession pat81 of *C. melo *L. ssp. *agrestis *(Naud.) Pangalo. The sources of RNA to construct each library were fruits of 15 and 46 days after pollination (dap), leaves, photosynthetic cotyledons inoculated with *Cucumber mosaic virus *(CMV), healthy roots and *Monosporascus cannonballus *Pollack et Uecker (the causal agent of melon vine decline) infected roots (Table 1). Approximately 3,700 sequences were determined from each library by single-pass 5' sequencing, except for the library prepared from CMV infected cotyledons for which approximately 6,600 sequences were determined, yielding a total of 33,292 raw sequences. Processing to eliminate vector sequences, low quality chromatograms and sequences of less than 100 base pairs (bp) gave rise to 29,604 good quality expressed sequence tags (ESTs) (Table 2) implying a cloning success of approximately 89%. The average edited length was 674 bp, and only a 6.4% of the sequences had less than 350 bp.

**Table 1:** Description of cDNA libraries

Name	Subspecies/cultivar/accession	Tissue/physiological condition
15d	Ssp. *melo *cv. "Piel de Sapo" T-111	Fruit 15 days after pollination
46d	Ssp. *melo *cv. "Piel de Sapo" T-111	Fruit 46 days after pollination
A	Ssp. *agrestis *accession pat81	Roots
AI	Ssp. *agrestis *accession pat81	Roots infected with *M. cannonballus*
CI	Ssp. *melo *var. cantaloupe accession C-35	Photosynthetic cotyledons infected with CMV
HS	Ssp. *melo *var. cantaloupe accession C-35	Leaves
PS	Ssp. *melo *cv. "Piel de Sapo" Piñonet torpedo	Roots
PSI	Ssp. *melo *cv. "Piel de Sapo" Piñonet torpedo	Roots infected with *M. cannonballus*

**Table 2:** EST statistics

Library	Raw sequences	Good-Quality ESTs	EST length	Singletons	Contigs	Unigenes	Redundancy (%)	Library-specific unigenes	Novelty (%)
15d	3,936	3,582	608.1 ± 175.2	1,009	1,930	2,939	18	1,100	37
46d	3,840	3,493	583.0 ± 161.1	1,000	1,854	2,854	18	1,063	37
A	3,936	3,666	700.0 ± 185.4	1,289	1,900	3,189	13	1,365	43
AI	3,647	3,255	756.3 ± 137.1	928	1,688	2,616	20	1,005	38
CI	6,605	5,664	651.4 ± 205.7	2,089	2,590	4,679	17	2,264	48
HS	3,648	3,012	669.3 ± 171.1	939	1,609	2,548	15	998	39
PS	3,840	3,377	679.9 ± 198.7	1,179	1,766	2,945	13	1,258	43
PSI	3,840	3,555	749.3 ± 156.2	1,279	1,826	3,105	13	1,363	44
	33,292	29,604		10,614	6,023	16,637			

**Figure 1:**
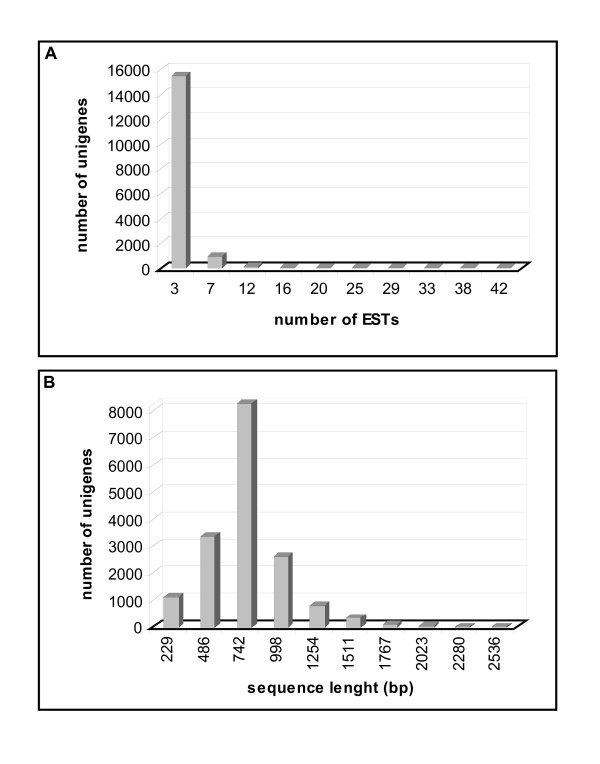
**Unigenes statistics**. *(A) *Distribution of melon ESTs among unigenes (contigs and singletons). *(B) *Size distribution of melon unigenes.

Clustering of the sequences using default parameters of the EST analysis pipeline EST2uni [43] yielded 6,023 tentative consensus sequences (also called contigs) and 10,614 unclustered sequences (also called singletons), with a total of 16,637 non-redundant sequences or unigenes (Table 2). All good quality ESTs were used for clustering, independently of the melon genotype of origin, because single nucleotide polymorphisms (SNPs) were expected among genotypes. The number of ESTs per unigene was between 1 and 44 (1 case), with an average of 1.8 ESTs per contig, as a high proportion of contigs (4,886 out of 6,023) contained less than 5 ESTs and contigs with more than 8 ESTs were scarce (Fig. 1A). Therefore, redundancy values were notably low (around 16%). The unigene length varied between 101 bp and 2,664 bp, averaging 751 bp (Fig. 1B). Library specific unigenes were about one third of the total for each library (Table 2). A second round of clustering yielded 14,480 unigene clusters, referred to as superunigenes. A web integrated database that contains all EST sequences, contig images and several tools for analysis and data mining has been created and named MELOGEN [44]. Codon usage was estimated using this EST collection. As expected, the codon usage of melon was very similar to that of Arabidopsis and other dicots. The preferred stop codon was UGA occurring in the 48% of the sequences. Suppression of the CG dinucleotide in the last two codon positions is very frequent in dicots, possibly as a consequence of methylation of C in the CG dinucleotide, resulting in an increased mutation rate [45]; in agreement with these data, the ratio XCG/XCC for melon was 0.52, very similar to the corresponding figure for tomato (0.58), pea (0.51), potato (0.48) and other dicots [45].

Libraries obtained from tissues inoculated with *M. cannonballus *were expected to contain sequences from the fungus. To estimate the proportion of sequences of fungal origin in these libraries, BLAST analyses against a database with plant and fungal sequences were carried out [46]. Only 56 sequences from these libraries were found to have a more significant similarity with fungal sequences than with plant sequences (Table 3). Consequently, these sequences were considered of fungal origin [46].

**Table 3:** ESTs showing significant similarity with fungal sequences

Library	Number of ESTs
15d	0
46d	0
A	2
AI	26
CI	0
HS	1
PS	1
PSI	30
Total	60

### SSRs and SNPs

We have analysed the nature and frequency of microsatellites or simple sequence repeats (SSRs) in the melon sequence dataset. A search for repeats of two, three or four nucleotides in the dataset yielded 1,052 potential SSRs. Approximately, 6% of the unigenes contained at least one of the considered SSRs motifs, with repeats of three nucleotides being prevalent (Table 4). The maximum and minimum lengths of the repeats were 68 and 17 nucleotides, respectively, and the average length was 26 nucleotides. The most common repeat among dinucleotides was, by far, the AG repeat, constituting the 83% (Table 4). Repeats of AT and AC dinucleotides followed, with approximately 9% and 7%, respectively. Among the trinucleotide repeats, the most frequent was AAG (66%, Table 4), and the least frequent was ACT (0.6%, Table 4). Among tetranucleotide repeats, the most frequent was AAAG (51%, Table 4). A high proportion of SSRs (29.5%) were found in open reading frames (ORFs), though an analysis of the localization of di-, tri- and tetranucleotides separately showed that di- and tetranucleotides localised preferentially in untranslated regions (UTRs), whereas trinucleotides localised in both, UTRs and ORFs (Table 5).

**Table 4:** Simple sequence repeats (SSRs) statistics*

Dinucleotide repeat	Number of di-pSSR	%
AG	205	83.3
AT	23	9.4
AC	18	7.3
Total	246	100

Trinucleotide repeat	Number of tri-pSSR	%

AAC	41	5.7
AAG	471	66.0
AAT	22	3.1
ACC	18	2.5
ACG	14	2.0
ACT	4	0.6
AGC	25	3.5
AGG	54	7.6
ATC	48	6.6
CCG	17	2.4
Total	714	100

Tetranucleotide repeat	Number of tetra-pSSR	%

AAAC	8	8.7
AAGG	7	7.6
AATC	4	4.3
AATG	3	3.3
AATT	2	2.2
ATCG	2	2.2
ACTC	1	1.1
AAGC	1	1.1
ACAT	1	1.1
AAAG	47	51.1
AAAT	16	17.3
Total	92	100

*The number of di-, tri- and tetranucleotide repeats identified in the melon database is shown for the complete set of putative SSRs (pSSRs).

**Table 5:** Localization of simple sequence repeats (SSRs) with respect to putative initiation and termination codons in the melon sequence dataset*

	Dinucleotide repeats		Trinucleotide repeats		Tetranucleotide repeats		All SSRs analyzed	
	
	No.	%	No.	%	No.	%	No.	%
	
5'-UTR	65	82.3%	121	51.7%	18	62.1%	204	59.6%
ORF	0	0.0%	99	42.3%	2	6.9%	101	29.5%
3'-UTR	13	16.5%	13	5.6%	9	31.0%	35	10.2%
Other^†^	1	1.3%	1	0.4%	0	0.0%	2	0.6%
Total	79	100.0%	234	100.0%	29	100.0%	342	100%

*Only full length melon unigenes were used for this analysis. Full length unigenes were automatically selected by checking the presence of the start codon by comparison of the 5'-terminal region of the unigene with the corresponding Arabidopsis gene used for annotation. ^†^Imprecise localization of the SSR with respect to putative initiation or termination codons.

Single nucleotide polymorphisms (SNPs) are the most abundant variations in genomes and, therefore, constitute a powerful tool for mapping and marker-assisted breeding. We initially identified in the melon sequence dataset 14,074 single nucleotide sequence variations and therefore potential SNPs (pSCH; Table 6) distributed in 4,663 contigs; however, these variations would include high-quality SNPs (pSNP) but also sequencing errors and mutations introduced during the cDNA synthesis step. Using more stringent criteria, these figures were substantially reduced: Putative SNPs were annotated only when the least represented allele was present in at least two EST sequences from the same genotype in a given contig and showing the same base change. Two accessions of the same cultivar (cv. "Piel de sapo") represented 47.3% of the sequences, but more than one half of the sequences were from two other more distant genotypes, the C-35 cantaloupe accession (29.3%) and the pat81 agrestis accession (23.4%). Thus, a total of 356 high-quality SNPs were found in 292 contigs, averaging 1.2 SNPs per contig. Transitions were much more common than transversions. There were 117 AG and 112 CT transitions compared with 28 AC, 37 AT and 33 GT transversions (Table 6). CG transversions were not detected. The MELOGEN database [44] includes a tool for designing oligonucleotide primers to amplify the region containing the polymorphism to generate the corresponding molecular marker.

**Table 6:** Single nucleotide polymorphisms (SNPs) statistics*

Variation		Number	%
pSNP transversions:		127	35.7
pSNP transitions:		229	64.3
pSCH transversions:		4,273	30.4
pSCH transitions:		9,801	69.6

Mutation	Number of pSNP	Mutation	Number of pSCH

A<->C	28	A<->C	859
A<->G	117	A<->G	5,860
A<->T	37	A<->T	1,897
C<->G	29	C<->G	431
C<->T	112	C<->T	3,941
G<->T	33	G<->T	1,086

*Type and number of transition and transversions are shown for putative single nucleotide variations in sequence (pSCH) and for putative high-quality single nucleotide polymorphism (pSNP) identified in the melon database.

### Functional annotation

In order to identify melon unigenes potentially encoding proteins with known function, we carried out a BLASTX analysis [47] of the sequence dataset against the databases listed in Table 7. Out of the 13,019 unigenes with a hit with proteins in databases, 11,431 (68.7%) unigenes showing an E value of ≤ 1e-10 were annotated. On the other hand, 31.3% of the unigenes did not show significant similarity to any protein in the databases and, therefore, were not annotated.

**Table 7:** Functional annotation statistics

**A**. Number of unigenes with BLAST hits		
Database*	Number of unigenes	%

Arabidopsis	11,724	70.5
Cucurbitaceae	5,340	32.1
Uniref90	11,893	71.5
Any database	13,019	78.3

**B**. Number of unigenes with HMMER hits		

Database*	Number of unigenes	%

Pfam	4,655	28.0

**C**. Number of unigenes with orthologue		

Database*	Number of unigenes	%

Arabidopsis	6,673	40.1

* Databases searched were: Arabidopsis [6,109]; Uniref90 [110,115]; Cucurbitaceae: all cucurbitaceae sequences available in the National Center for Biotechnology Information (NCBI); any database: results using Arabidopsis, cucurbitaceae and Uniref90 databases all together; Pfam [49,112].

**Figure 2:**
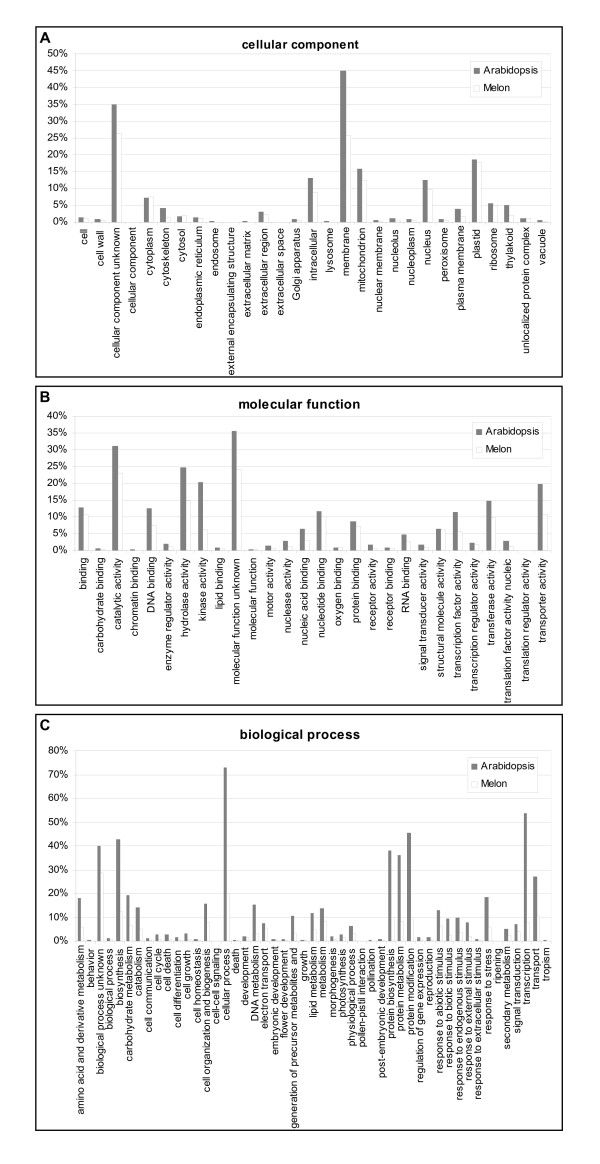
Distribution of melon and Arabidopsis unigenes according to the Gene Ontology scheme for functional classification of gene products.

Additionally, we performed a functional classification of the unigenes following the Gene Ontology scheme. Gene Ontology provides a structured and controlled vocabulary to describe gene products according to three ontologies: molecular function, biological process and cellular component [48]. To do that, we added GO terms based on the automated annotation of each unigene using the Arabidopsis database [6]. A summary of the results with the percentage of unigenes annotated in representative categories corresponding to the GO slim terms [48] is shown, as well as a comparison of the distribution of melon and Arabidopsis unigenes (Fig. 2). The distributions of melon and Arabidopsis unigenes follow similar tendencies, suggesting that the melon dataset is representative of the whole melon transcriptome. In total, 9,402 unigenes could be mapped to one or more ontologies, with multiple assignments possible for a given protein within a single ontology. A high percentage of unigenes in both species was classified as "unknown function". Out of the 9,791 assignments made to the cellular component category, 25.8% corresponded to membrane proteins and 17.8% to plastidial proteins (Fig. 2A). Under the molecular function category, assignments were mainly to catalytic activity (23.0%) and to hydrolase activity (14.7%) (Fig. 2B). The distribution of unigenes under the biological process category was more uniform, with 19.9% of assignments to cellular process and 12.7% to biosynthesis (Fig. 2C).

We have also identified 6,673 (40.1%) melon unigenes with an ortholog in the Arabidopsis database, and a HMMER motif has been assigned to 4,655 (28.0%) unigenes by comparisons with the Pfam database [49] (Table 7). All these results are compiled in the MELOGEN database, which also contains direct links to the databases used to carry out analyses.

### Genes potentially encoding microRNAs

**Figure 3:**
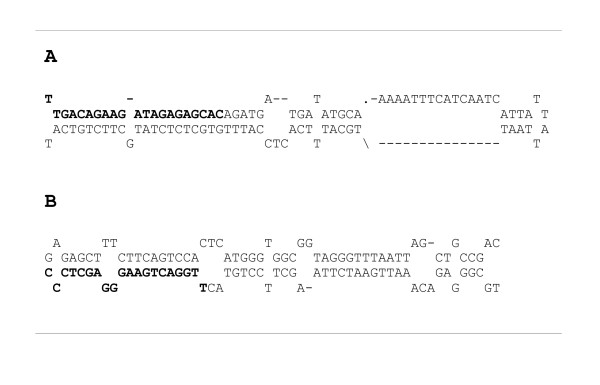
**Potential precursors of melon microRNAs**. *(A) *Stem loop sequence of putative precursor miRNA corresponding to unigene bCI_04-H02-M13R_c. *(B) *Stem loop sequence of putative precursor miRNA corresponding to unigene b15d_24-H05-M13R_c. The mature miRNA sequences are shown in bold.

Central to RNA silencing are small RNA molecules (sRNAs) that can arise from endogenous or exogenous sources from precursors with double-stranded RNA (dsRNA) pairing. One class of such sRNAs are microRNAs (miRNAs), which originate from endogenous long self-complementary precursors that mature in a multi-step process involving many enzymes [50,51]. Recently, a comprehensive strategy to identify new miRNA homologs in EST databases has been developed [52,53]. We have followed this strategy to identify potential melon miRNAs. A total of 20 ESTs that contained homologs to miRNAs in the microRNA Registry database [54] were identified and grouped into 12 contigs and, after manual inspection of secondary foldback hairpin structure, 5 unigenes were selected (Table 8). Contig sequences varied between 536 and 840 nucleotides long, and had negative folding free energies of -206.8 to -160.8 kcal mol^-1 ^(Table 8) according to MFOLD [55], which are in the range of the computational values of Arabidopsis miRNA precursors [52]. Their predicted secondary structures showed that there were at least 16 nucleotides paired between the sequence of the potential mature miRNA and its opposite arm (miRNA*) in the corresponding hairpin structure (Fig. 3). The location of the potential miRNAs varied among ESTs, 4 were found in the sense orientation of the EST, 1 was found in the antisense orientation. We have also searched for potential targets of the potential miRNAs in the melon EST dataset, identifying 3 of them (Table 8). However, minimal folding free energy indexes (MFEIs) [53] were below the -0.85 cut-off value proposed by Zhang et al. [53] only for m12 (Table 8). Potential melon miRNA m12 has a precursor of 536 nt in length and codes for a melon ortholog of the Arabidopsis miR319. miR319 targets a transcription factor of the TCP family [56,57]; in the melon dataset, an ortholog of this Arabidopsis gene has been found in a unigene annotated as a TCP transcription factor. In this case, the melon miRNA and its potential target have a pattern of paired/non-paired bases between the target and the miRNA identical to the corresponding target-miRNA pattern in Arabidopsis (data not shown).

**Table 8:** Potential melon miRNAs

Name	Potential mature miRNA sequence (5'->3')*	Melogen unigene^†^	Precursor folding free energy (kcal mol^-1^)^§^	MFEI (kcal mol^-1^)^Δ^	miRNA family	Potential target in melon
m2	ugaagcugccagcaugaucu	bCL2353Contig1	-206.8	-0.67	miR167	--
m4	ugauugagccgugccaauauc	bPSI_40-F10-M13R_c	-195.0	-0.66	miR171	bHS_39-C12-M13R_c
m7	ucggaccaggcuucauucccc	bA_31-D02-M13R_c	-160.8	-0.70	miR166	--
m8	uugacagaagauagagagcac	bCI_04-H02-M13R_c	-188.2	-0.74	miR157	bCI_30-A09-M13R_c
m12	uuggacugaagggagcucccu	b15d_24-H05-M13R_c	-163.6	-0.86	miR319	bCL2243Contig1

*Nucleotide sequences correspond to potential mature miRNA sequences as deduced from BLAST searches using known plant miRNA sequences [54] and analysis of secondary structure predictions [52]. ^†^Accession numbers (MELOGEN database) of potential precursors of melon miRNAs are given. ^§^Computational values of folding free energies have been calculated using MFOLD 3.1 [55]. ^Δ^Minimal folding free energy indexes (MFEIs) have been calculated as described by Zhang et al. [53].

### Genes potentially encoding pathogen resistance and fruit quality traits

Pathogens affect severely the productivity of melon crops. Three of the cDNA libraries sequenced here correspond to pathogen-infected tissues and, thus, should contain transcripts from genes whose expression is induced in response infection. We have carried out a bioinformatics search for homologs of genes involved in pathogen resistance response (see [58] for a review) and virus susceptibility [59-61], finding among them at least one melon ortholog to the Arabidopsis FLS2 receptor [62], several unigenes potentially encoding disease resistance proteins as well as mitogen-activated protein kinases, homologs to translation initiation factors constituting potential virus susceptibility factors, etc. [see Additional file 1].

Fruit development and ripening are the most important processes determining the fruit quality traits of fleshy fruits like melon. At present most of the molecular and genetic data available about fruit development and ripening come from tomato [63,64] and Arabidopsis [65,66]. In recent years, several genes and quantitative trait loci controlling fruit quality traits have been described in melon [67,68]. As for developmental processes, homologs to genes involved in melon fruit development, ripening and quality have been found in the melon dataset. These include several MADS-box genes, homologs to the *fw2.2 *and *ovate *QTLs [69,70], several homologs to members of the SBP-box gene family to which the major tomato ripening gene *COLORLESS NON-RIPENING *belongs [71], several ACC synthase and ACC oxidase genes, unigenes from several cell wall-metabolism enzymes, etc [see Additional file 1].

### Expression analysis of selected ESTs by Real-Time-qPCR

The accumulation of transcripts for 20 selected genes was analyzed by reverse transcription Real-Time-qPCR. ESTs for this analysis were preferentially chosen among those showing significant similarity with genes related to response to infection and fruit quality characteristics in melon and other species, and included *CTL1, EIF4A-2, EIF4E, EIN4, GA2OX1, HSP101, HSP70, IAA9, LSM1, LUT2, NCBP, SVP, HIR, TCH4, TIP4, TOM1, TOM2A, TOM3, UGE5 *and *WRKY70 *(Table 9). Preliminary experiments were carried out to choose between *GAPDH *and *CYCLOPHILIN *(*CYP7*) RNAs as endogenous controls; results showed that the *CYP7 *RNA levels varied the least among treatments (data not shown) and, therefore, transcript accumulation levels were expressed relative to *CYP7 *RNA levels.

**Table 9:** Transcripts selected from the database for gene expression analysis by Real Time qPCR

Gene	Melogen unigene	Sequence length (bp)	Arabidopsis *locus*	Aminoacid similarity (%)	Annotation (HMMR domain)
*CTL1*	bCL1465Contig1	1,387	AT1G05850	66.8	Chitinase-like protein 1, similar to class I chitinase (Glyco_hydro_19)
*CYP 7*	bCL3337Contig1	801	AT5G58710	78.4	Peptidyl-prolyl cis-trans isomerase, cyclophilin
*EIF4A-2*	bCL2906Contig1	1,146	AT1G54270	92.6	Eukaryotic translation initiation factor 4A-2 (DEAD)
*EIF4E*	bCL4710Contig1	815	AT4G18040	77.5	Eukaryotic translation initiation factor 4E 1 (IF4E)
*EIN4*	bCL1742Contig1	829	AT3G04580	55.7	Ethylene receptor (Response_reg)
*GA2OX1*	bCL1313Contig1	1,454	AT1G78440	59.9	Gibberellin 2-oxidase (2OG-FeII_Oxy)
*HSP101*	bCI_38-F11-M13R_c	726	AT1G74310	73.4	Heat shock protein 101 (AAA_2)
*HSP70*	bPSI_41-D06-M13R_c	819	AT5G09590	86.8	Heat shock protein 70/HSC70-5 (HSP70)
*IAA9*	bCL1341Contig1	1,672	AT5G65670	51.2	Auxin-responsive protein/indoleacetic acid- induced protein 9 (AUX_IAA)
*LSM1*	bHS_37-F11-M13R_c	766	AT3G14080	72.4	Small nuclear ribonucleoprotein/snRNP, putative/Sm protein, putative, similar to U6 snRNA-associated Sm-like protein
*LUT2*	bCL3563Contig1	1,082	AT5G57030	75.5	Lycopene epsilon cyclase (Lycopene_cycl)
*NCBP*	bCL183Contig1	1,081	AT5G18110	85.3	Novel cap-binding protein (IF4E)
*SVP*	bCL2852Contig1	826	AT2G22540	54.9	Short vegetative phase protein, MADS box transcription factor related cluster (SRF-TF)
*HIR*	bCL144Contig1	1,266	AT1G69840	89.7	Band 7 family protein, strong similarity to hypersensitive-induced response protein (Band_7)
*TCH4*	bCL1212Contig1	1,228	AT5G57560	65.9	Xyloglucan:xyloglucosyl transferase/xyloglucan endotransglycosylase/endo- xyloglucan transferase/TCH4
*TIP4*	bA_23-B12-M13R_c	751	AT2G25810	50.5	Tonoplast intrinsic protein (MIP)
*TOM1*	bCL4416Contig1	780	AT4G21790	75.4	Transmembrane protein-related/TOM1 (DUF1084)
*TOM2A*	bCL3115Contig1	1,113	AT1G32400	60.1	Senescence-associated family protein
*TOM3*	bPSI_36-E03-M13R_c	857	AT1G14530		Tobamovirus multiplication protein 3/THH1 (DUF1084)
*UGE5*	bCL1153Contig1	1,313	AT4G10960	70.9	UDP-glucose 4-epimerase/UDP-galactose 4- epimerase/Galactowaldenase (Epimerase)
*WRKY70*	bA_25-B01-M13R_c	889	AT3G56400	42.1	WRKY family transcription factor, DNA-binding protein (WRKY)

**Figure 4:**
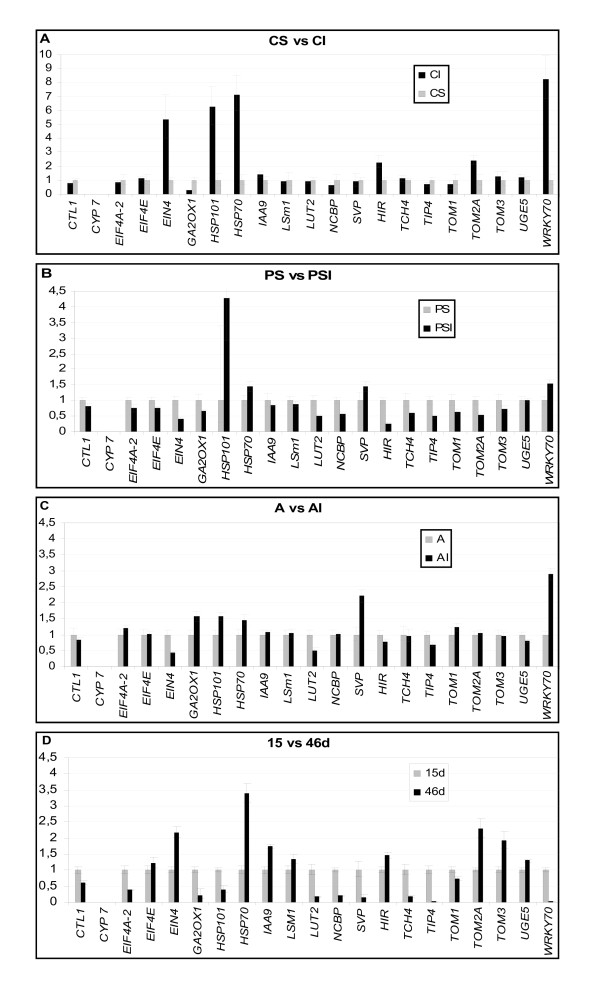
**Transcripts analyzed by Real Time qPCR**. *(A) *Pattern of transcripts accumulation in CMV-infected melon cotyledons (CI) relative to that of healthy cotyledons (CS). *(B) *Pattern of transcripts accumulation in *M. cannonballus *infected roots of *C. melo *L. cv. "Piel de sapo" (PSI) relative to that of healthy roots (PS). *(C) *Pattern of transcripts accumulation in *M. cannonballus *infected roots of *C. melo *L. ssp. *agrestis *(AI) relative to that of healthy roots (A). *(D) *Pattern of transcripts accumulation in fruits of 15 days after pollination of *C. melo *L. cv. "Piel de sapo" (15d) relative to that of fruits of 46 days after pollination (46d). cy: cyclophilin endogenous control; see Table 9 for the rest of genes.

Figure 4A illustrates the alteration of the RNA accumulation levels of selected genes that occurred in photosynthetic cotyledons after CMV infection. A significant increase in the level of transcripts from *HSP101, HSP70, HIR, TOM2A, WRKY70 *and *EIN4 *was observed; for *HSP101, HSP70, WRKY70 *and *EIN4*, transcript accumulation levels in inoculated cotyledons were up to five times greater than in uninoculated controls (Fig. 4A). All of these genes, except *TOM2A*, have been shown to be responsive to virus infection in other hosts [72-74]. Notably, the expression of *EIF4E*, known to be required for MNSV multiplication [27], remained unaltered. A shutoff of host gene expression also occurs in association with virus infection [75]; for the set of genes analysed here, only *GA2OX1 *and *NCBP *responded to CMV infection with a reduction in the accumulation of their transcripts.

The response of selected genes in roots inoculated with *M. cannonballus *was analysed in melon genotypes known to be susceptible (cultivar "Piel de sapo"; Fig. 4B) and partially resistant (accession pat81 of *C. melo *L. ssp. *agrestis*; Fig. 4C) to the infection by this fungus. The patterns of transcript accumulation resulted clearly different for both genotypes. For pat81 (resistant), transcription factors *WRKY70 *and *SVP *increased their expression between 2 and 3 times after inoculation; other stress-inducible genes (*HSP101, HSP70*) showed only a moderate increase (Fig. 4C). For "Piel de sapo" (susceptible), accumulation of *WRKY70 *and *SVP *transcripts only increased about 1.5 times after inoculation whereas the expression of *HSP101 *showed a marked increase (Fig. 4B). It is also worth noting the differential response of the *GA2OX1 *gene in the two genotypes. Expression of *GA2OX1 *increased about 1.5 times in pat81 roots after the *M. cannonballus *attack, whereas it decreased in "Piel de sapo" roots after fungal infection (compare Figs. 4B and 4C).

Comparison of patterns of transcript accumulation at two stages of fruit development showed increased levels of gene expression for 9 of the analysed genes. This was particularly evident for *HSP70, TOM2A, TOM3, EIN4 *and *IAA9*. In contrast, decreased levels of transcript accumulation were observed for the other 11 genes.

## Discussion

In this paper we provide an initial platform for functional genomics of melon by the identification of more than 16,000 unigenes assembled from almost 30,000 ESTs sequenced from 8 melon cDNA libraries. It is probably premature to estimate the proportion of melon genes represented in this dataset, but based on available data for other plant species (i.e. Arabidopsis and rice), it is likely that the melon unigene set characterised here represents approximately between half and one-third of the number of expressed, protein coding genes of melon. Libraries were constructed from various tissue types, but with a bias towards fruit development and pathogen-infected tissues. Data from these libraries will become a useful resource of genes for experiments aimed at understanding important processes involved in fruit development and resistance to viral and fungal pathogens. Also, data presented here provide an important tool for generating markers to saturate melon genetic maps.

In contrast to typical EST gene-sampling strategies reported previously, we have found a low degree of redundancy in the sequences determined. The process of clustering reduced the number of sequences to 56%, from 29,604 good quality ESTs to 6,023 contigs and 10,614 singletons. Contigs with more than 8 ESTs were scarce, the majority of them being formed by 3 or 2 ESTs. Redundancy of the sequences derived from each library ranged from 13% to 20%, with singletons constituting approximately one third of the unigenes determined per library. This low redundancy is probably due to the success of the normalization process, responsible for the suppression of superabundant transcripts specific for a given tissue or condition. Normalization precludes *in silico *analysis of gene expression, but greatly increases the number of unigenes that can be determined by reducing redundancy [76]. Here we have used a recently described normalization protocol which is based on the cleavage of DNA or DNA-RNA duplexes by a specific DNase [77]; this process, in our hands, has proven simple, reproducible and efficient. Another factor that has contributed to the low redundancy values obtained has been the sequencing of libraries from very distinct tissues. Thus, the number of library specific unigenes was about one half of the total number of unigenes contributed by each library, suggesting that further sequencing of the libraries still has the potential to provide a good number of new, non-redundant sequences.

cDNA sequences are a useful source of SSRs, which are excellent molecular markers due to their high degree of polymorphism. A common feature of cDNA sequences obtained from plants is the high frequency of SSRs that they contain [36]. We have identified more than 1,000 potential SSRs in the melon dataset, with approximately 6% of the melon unigenes containing di-, tri- or tetranucleotide repeats. A clear bias toward AG and AAG repeats existed, that account for 67% of the SSRs. In contrast, the GC repeat was not found in the melon dataset. A similar bias toward AG and against CG repeats has been identified in Arabidopsis and other plant species [40,78]. As proposed at least in one other instance [40], this may be due to the tendency of CpG sequences to be methylated [79], which potentially might inhibit transcription. Another interesting feature of melon SSRs relates to their pattern of localization with respect to putative initiation and termination codons. It is known that the UTRs of transcribed sequences are richer in SSRs than coding regions, particularly at the 5'-UTRs [36,40]. However, in the melon dataset, a high proportion of SSRs (29.5%) were found in ORFs. An analysis of the localization of di-, tri- and tetranucleotide repeats separately showed that di- and tetranucleotides were preferentially located in UTRs, whereas trinucleotides localised in both, UTRs and ORFs, consistently with maintenance of the ORFs coding capacity. Thus, the prevalence of trinucleotide repeats in the melon dataset (71%) explains this result.

We identified in the melon sequence dataset 356 high-quality SNPs. Since non-redundant sequences analysed here encompassed 4.5 Mb, one SNP was found every 12,000 pb of sequence. This small figure is probably due to the limited number of melon genotypes used and the low redundancy found among libraries. In fact, when the frequency of SNPs is computed in relation to the length and number of contigs containing SNPs, the corresponding value (one SNP in every 616 bp of sequence) is of the same order of magnitude as values previously calculated for melon (441 bp; [80]) and other plant species [40]. With the advent of high-throughput detection systems, the SSRs and SNPs identified here will constitute an important resource for mapping and marker-assisted breeding in melon and closely related crops.

As an approach to the function of melon unigenes, we carried out a bioinformatics analysis based on BLASTX and matches with the Pfam database [49]. The proportion of melon unigenes with no similar sequences in databases was quite high, suggesting that the melon dataset may encompass an important number of melon-specific sequences. However, the proportion specific sequences might be overestimated because blasting has been made with unigene sequences, which in many cases do not cover the complete length of the transcript. We performed a functional classification of the unigenes following the Gene Ontology scheme, which is one of the more versatile and complete systems for functional classification [48]. A comparison of the distributions of melon and Arabidopsis unigenes in GO categories showed that both followed similar tendencies, suggesting that the melon dataset is representative of the whole melon transcriptome. This is remarkable, as the number of different libraries sequenced has been relatively small; again, this is probably due to the success of the normalization process. We have also carried out specific searches for genes involved in pathways of particular relevance in melon, such are resistance response and fruit development, identifying a remarkable number of melon candidates. For example, an ortholog of the flagellin receptor FLS2 from Arabidopsis [62] has been identified, together with 163 candidate RLKs that may have critical roles in pathogen recognition or diverse signalling processes. Similarly, up to 8 MADS-box gene homologs with potential roles in development have been found in the melon dataset. Moreover, a bioinformatics approach [52,53] allowed the identification of potential precursors of melon miRNAs together with several potential targets in the melon dataset. This finding opens the door to biotechnology approaches based on the use of artificial miRNAs to specifically silence melon genes [81,82].

The transcript accumulation analysis for the 20 selected genes revealed important changes in gene expression associated with pathogen infection and fruit development. For virus infection, the accumulation of transcripts remained unaltered for 12 genes, but showed a significant increase for 6 genes and a decrease for 2. Among the set of genes analysed, *TOM2A *and *EIF4E *were known to code for virus susceptibility factors [27,83]; the expression of *TOM2A *was increased after CMV infection, consistently with its requirement by the virus, but this was not the case for *EIF4E*. Different hypotheses can explain this result: since EIF4E is an abundant, housekeeping protein, increased expression may not be essential for virus multiplication; alternatively, CMV may not use EIF4E in melon or may use a factor coded by a different member of the 4E family; it may also be that timing of the sampling for this experiment was not appropriate to detect such an effect, as requirement of EIF4E might occur very early during virus multiplication. In the cases of infection of susceptible ("Piel de sapo") and resistant (pat81) melons by *M. cannonballus*, more extensive alterations in gene expression seemed to occur in the susceptible than in the resistant accession. Significantly, for the susceptible accession, stress responsive genes (e.g. *HSP101*) appeared to be maximally induced, whereas for the resistant accession, a gene encoding a WRKY70 transcription factor, potentially involved in resistance response, was induced to high levels. Significantly, expression of *GA2OX1 *increased about 1.5 times in pat81 after the *M. cannonballus *attack, whereas it decreased in "Piel de sapo". *GA2ox *is a major gibberellin (GA) catabolic enzyme, with an important role in controlling GA levels in plants. Hormones control many plant developmental processes, and strong evidence indicates that hormone signalling is involved in the regulation of root growth and architecture [84,85]. The differential response of the *GA2OX1 *gene in the two melon genotypes is consistent with an enhanced root growth in pat81 after infection [86]. Notably, other genes involved in hormone-mediated signalling pathways, such as the *IAA9 *gene, did not show such differential response to *M. cannonballus *infection in both genotypes. In the case of fruit development, differences in the expression of selected genes between immature and ripening fruits appeared to be even sharper than in the cases of healthy and pathogen-infected tissues. Specific roles during fruit development for *HSP70, TOM2A *and *TOM3 *have not been identified, though an increased expression has been shown at least in the case of *TOM2A *in tomato [87]. The ethylene receptor gene *EIN4 *showed a two-fold increase in expression. *EIN4 *is the ortholog of Arabidopsis *EIN4 *and tomato *LeETR4 *[88,89]. In tomato, *LeETR4 *is also highly expressed in ripening fruit, suggesting that it responds by modulating ethylene signalling during ripening [63]. The MADS-box gene (*SVP*) showed about a four-fold decrease in expression. This gene is the ortholog of tomato *JOINTLESS*, which specifies the abscission zone in tomato. In tomato fruit microarray hybridizations, the expression of *JOINTLESS *also decreased from 7 to 57 DAP [87], in agreement with our data for melon. The lycopene epsilon cyclase (*LUT2*) and xyloglucan endotransglycosylase (*TCH4*) genes showed an approximately four-fold decrease in expression during melon fruit development. These findings fit with the patterns of expression of these genes in tomato, where their transcript levels decrease to a non-detectable level in the ripe fruits [90,91].

## Conclusion

In summary, this collection of ESTs represents a substantial increase on the information available for melon. The dataset contains SSR and SNP markers that can be used for breeding, as well as a significant number of candidate genes that can be experimentally tested for their roles in various important processes. This set of genes constitutes also the basis for a microarray for melon that is being used in experiments to further analyse fruit development and maturation and responses to pathogen infections.

## Methods

### Plant material

The cDNA libraries were prepared using material from four different melon genotypes: the line T-111 (Semillas Fitó, Barcelona, Spain), which corresponds to a Piel de Sapo breeding line, the Piel de Sapo cultivar "Piñonet torpedo" (Semillas Batlle, Barcelona, Spain), the accession C-35 of the germplasm collection of La Mayora-CSIC (EELM-CSIC, Málaga, Spain), which corresponds to a cantaloupe-type of melon, and the accession pat81 of *C. melo *L. ssp. *agrestis *(Naud.) Pangalo maintained at the germplasm bank of COMAV (COMAV-UPV, Valencia, Spain) (Table 1). Seeds of line T-111 were germinated at 30°C for two days and plants were grown in a greenhouse in peat bags, drip irrigated, with 0.25-m spacing between plants. Fruits of 15 and 46 days after pollination were collected and mesocarp tissues were recovered and used for RNA extractions. Root samples were from Piel de sapo and pat81 plants, both healthy and inoculated with *M. cannonballus*. Piel de sapo is fully susceptible to the infection by this fungus whereas pat81 has been shown to be partially resistant [92,93]. Seeds were pre-germinated in Petri dishes. After 4 days, seedlings were transplanted to 0.5-l pots filled with sterile soil substrate and grown in a greenhouse (20–35°C, 60–85% relative humidity). Inoculations were carried out by adding 50 colony-forming units (CFU) of *M. cannonballus *per gram of sterile soil as described by Iglesias et al. [94]. Fourteen days after inoculation, healthy and inoculated roots were collected for RNA extraction. The presence of the fungus and the infection levels were assessed by real-time quantitative PCR as described by Picó et al. [95]. CMV infected cotyledons were collected from plants of the C-35 accession. In this case, seeds were pregerminated in Petri dishes for 24 h at 28°C in the dark, planted in 0.5-l pots and maintained in an insect-proof green house (20–28°C, 45 to 85% relative humidity) for 6 to 7 days, until the first true leaf started emerging. At this stage, cotyledons were mechanically inoculated with CMV following standard procedures [96]. Inoculated cotyledons were harvested 4 days after inoculation and used for RNA extractions. Dot-blot hybridisation [97] was used to check infection by CMV. Plants of the C-35 accession were also used for collecting healthy leaves. Plants were maintained in the greenhouse for 21 days, and second and third leaves above cotyledons were harvested for RNA extractions.

### Construction of cDNA libraries and EST sequencing

Total RNA was prepared as described by Aranda et al. [98]. Poly(A^+^) RNA from total RNA was purified using MicroPoly(A^+^) Purist (Ambion, Austin, TX, USA), a cellulose-oligo(dT)-based method. Integrity and quality of both total and poly(A^+^) RNA were tested by gel electrophoresis. cDNA libraries were constructed with the SMART cDNA Library Construction kit (Clontech, Mountain View, CA, USA), using a modified primer to include a *Sfi *I enzyme restriction site. A normalization step was carried out with TRIMMER kit (Evrogen, Moscow, Russia). After normalization, a cDNA fractionation step was performed with SizeSep 400 Spun Columns (Amersham Biosciences, Buckinghamshire, England). cDNA was digested with *Sfi *I, generating *Sfi *IA-*Sfi *IB cohesive ends for directional cloning into a modified version of BlueScript SK plasmid vector (Stratagene, La Jolla, CA, USA). Ligation products were transformed into *E. coli *electrocompetent cells DH10B (Invitrogen, Carlsbad, CA, USA) by electroporation. The titer of the libraries was evaluated by plating an aliquot on LB agar plates with ampicillin at 100 μg ml^-1^. Only libraries of 10^5 ^cfu ml^-1 ^or more were considered as acceptable. Prior to large scale sequencing, the average insert size was estimated by restriction analyses of 24 plasmid DNA minipreps per library from randomly picked colonies.

Sequencing was carried out from the 5'-end of the inserts without library amplification using the universal M13 reverse primer. An external custom service was contracted for this task (Macrogen Inc., Seoul, Korea). Approximately 6,000 clones were sequenced from the CI library, and 3,500 clones were sequenced from each of the other libraries (Table 2). Sequences obtained in this work can be found in GenBank [accession numbers AM713476 to AM743079] and MELOGEN [44].

### Bioinformatics

EST sequences were automatically trimmed, clustered and annotated using the EST2uni analysis pipeline [43]. EST2uni compromises the analysis pipeline written in PERL [99], a database (MySQL) [100] and a web site to browse the results coded in PHP [101]. Thus, for the EST pre-processing step, base calling was performed with Phred [102], low quality regions and vector sequences were trimmed with Lucy [103], and repeats and low complexity regions were masked with RepeatMasker [104] and Seqclean [105]. Further vector contamination was also eliminated with Seqclean using NCBI's UniVec [106]. High-quality EST sequences were then assembled to obtain the unigene set using Tgicl [105].

Detection of SSRs was performed using Sputnik [107]. Putative SNPs were annotated when the least represented allele was present in two EST sequences or more. ORFs were predicted in the ESTs with the aid of the ESTScan software [108].

For functional annotation, comparisons against the Arabidopsis (TAIR) [109] and Uniref [110] databases were carried out using BLASTN or BLASTX for nucleotide or protein sequences, respectively. Functional domains were searched with HMMPFAM [111] using the Pfam database [112]. The Gene Ontology (GO) classification [48] was derived from the BLASTN results against the Arabidopsis proteome. Also, a bi-directional BLASTN comparison was performed in order to obtain a set of putative orthologs with Arabidopsis. Finally, a set of superunigenes was obtained grouping different unigenes with the same expected mRNA target, as judged by extensive sequence overlapping.

To assess codon usage, we generated a set of melon sequences predicted to contain full-length coding regions. These sequences were subjected to BLASTX and, after manual inspection, sequences showing a high similarity to Arabidopsis proteins were selected to ensure that no sequences containing frame-shift errors were included in the analysis. From this smaller dataset, which included 588 sequences, ORFs were defined and a codon usage table was created. Codon usage was calculated from sequences using the GCUA program [113]. All codons were found in the dataset, with the least frequent codon represented 134 times.

To identify potential melon miRNAs, the 33,292 melon ESTs were subjected to a BLAST search against mature sequences of known miRNAs from the miRNA Registry Database (released January 2007) [54] using BLASTN [47]. ESTs with only 0–1 mismatched nucleotides with known miRNAs were considered. Selected ESTs were subjected to a BLAST search against protein databases in order to remove potential protein-coding sequences. ESTs pertaining to the same melon unigene of the MELOGEN database were grouped. The secondary structures of the unigenes encoding potential miRNA precursors were predicted with the web-based tool MFOLD [55], using default parameters. In each case, only the lower energy structure was selected for visual inspection, as previously described [52,114]. In order to select unigenes with perfect or near-perfect secondary foldback hairpin structures, only sequences with a maximum size of 3 nucleotides for a bulge in the miRNA sequence and with at least 16 paired nucleotides between the mature sequence and the opposite arm were considered as potential miRNA candidates. In addition, the minimal folding free energy index (MFEI) for each sequence was calculated following Zhang et al. [53].

### Gene expression analyses

Real time quantitative PCR was performed with an AB 7500 System (Applied Biosystems, Foster City, CA, U.S.A) to quantify mRNA corresponding to some transcripts of interest, in the tissues and physiological conditions used for library construction. Twenty ESTs representing these transcripts were chosen from the database and used to generate gene-specific primers (Table 10) with Primer Express Software (Applied Biosystems). The chemistry used for PCR product detection was the Power SYBR green dye (Applied Biosystems) and ROX as passive reference. *CYCLOPHILIN *served as endogenous control (sequence extracted from the database), ΔΔCt was the method of calculation to perform relative quantification, and three technical replicates were carried out and considered for statistical analysis. Melting curves analyses at the end of the process and No Template Controls (NTC) were carried out to ensure product-specific amplification and no primer-dimer quantification. A control reaction as for reverse transcription but without the enzyme was performed to evaluate genomic DNA contamination.

**Table 10:** Primer sequences for Real Time-qPCR analysis of transcript accumulation

Gene	Primer sequence (5'->3') Forward	Reverse
*CTL1*	tgggccatgttggctctaag	ctcccgtgacaactccatca
*CYP 7*	cgatgtggaaattgacggaa	cggtgcataatgctcggaa
*EIF4A-2*	ttcccgaggtttcaaagatca	ccaatgcttctggtggcact
*EIF4E*	ttcggttccttcccttccat	ccgccgatgtagctttcatc
*EIN4*	tgcaacgtgactgctgtttct	tctggcatgtgaagatccaaga
*GA2OX1*	tagggcaaatcggttagcga	ccaaatgcaaaccgattgaa
*HSP101*	aacgtatggtgcggattgaca	tccaccttcatggtatccaacat
*HSP70*	gctgaggcgtaccttggaaa	atcctgccagcatcctttgt
*IAA9*	gacggaaagccaggttcaag	cccctccatactcactttcacaa
*LSM1*	ctacttcgagatgggcggaa	tcaccaacgatcaccctttca
*LUT2*	gctggcgtggaacactcttt	cgaatgccttcaatgtccagt
*NCBP*	cgtcggtctgcttaatttgca	cgcctacgaactccattgaca
*SVP*	cgaggcaggtcacgttctct	agagaagacgaggagcgcaa
*HIR*	tgacgggctcagagacagtg	gttccagggacgttttcagc
*TCH4*	ggagggtagccttgagggaat	ctggacattgctcgacaacaa
*TIP4*	tccttgctggtgtcggatc	cgtttgccaataacgcattg
*TOM1*	gggagaaggaagaaacttcatgag	gcgtcaaaagcggataaagc
*TOM2A*	ctcctcagcagccgaagaaa	tggacccgctaaaacaccac
*TOM3*	aatggagttcgggctgttgt	aaggcaaggcttggcatgt
*UGE5*	gcgaaagtgtccaaaagcca	acacaagctttttgcatccgt
*WRKY70*	ggattgctcctggcctgac	tcggctgcttttcttcgatc

## Authors' contributions

Daniel Gonzalez-Ibeas prepared RNAs for two libraries, constructed the eight libraries, carried out the gene expression analysis by Real-Time-qPCR and participated in the bioinformatics analyses and in the drafting of the manuscript. José Blanca carried out the bioinformatics analyses, EST database and web page, and participated in the drafting of the manuscript. Cristina Roig and Belén Picó prepared RNAs for the root libraries and participated in the drafting of the manuscript. Mireia González-To, Wim Deleu and Jordi Garcia-Mas prepared RNAs from melon fruits and participated in the drafting of the manuscript. Pere Puigdomènech is the main coordinator of The MELOGEN Project and participated in the conception of the study together with Pere Arús, Fernando Nuez, Jordi Garcia-Mas and Miguel A. Aranda. Miguel A. Aranda is the principal investigator or this work, supervised it and wrote the manuscript. All authors read and approved the final manuscript.

## Supplementary Material

Additional file 1 Genes potentially encoding pathogen resistance and fruit quality traits. Genes were identified in the melon data set by comparison with the Arabidopsis database [6,109]. A brief description, the corresponding Arabidopsis locus and the HMMR domain identified are given for each unigene. Click here for file
