# Supplementary material for: MELOGEN: an EST database for melon functional genomics
Source: BMC Genomics. 2007 Sep 3;8:306. doi: 10.1186/1471-2164-8-306 (PMC2034596; doi:10.1186/1471-2164-8-306)
Supplement: Additional file 1 — Genes potentially encoding pathogen resistance and fruit quality traits. Genes were identified in the melon data set by comparison with the Arabidopsis database [6,109]. A brief description, the corresponding Arabidopsis locus and the HMMR domain identified are given for each unigene. [file 1471-2164-8-306-S1.pdf]

Additional file 1

Genes potentially encoding pathogen resistance and fruit quality traits. Genes were identified in the melon data set by comparison with the Arabidopsis database [6]. A brief description, the corresponding Arabidopsis locus and the HMMR domain identified are given for each unigene.

gene name[a]	description	Arabidopsis locus	Melogen unigene	HMMR domain
OFP6	ovate protein	AT3G52525	bCL2403Contig1	--
OFP1	ovate protein	AT5G01840	bCL5705Contig1	--
OFP2	ovate protein	AT2G30400	bCL1523Contig1	DUF623
OFP13	ovate protein	AT5G04820	bCL5616Contig1	DUF623
F10B6_27	ORFX homolog1 (fw2.2 encoding ORF), expressed protein, similar to PGPS/D12	AT1G14870	bCL2140Contig1	--
F9I5_19	ORFX homolog 2 expressed protein, similar to PGPS/D12	AT1G52200	bCL906Contig1	PLAC8
--	expressed protein, low similarity to PGPS/D12	AT2G40935	bAI_04-D10-M13R	--
SPL3	SPB-box gene, identical to squamosa-promoter binding protein like 3	AT2G33810	bCL2523Contig1	--
MBA10_13	SPB-box gene, similar to squamosa promoter binding protein-like 9	AT5G50570	bPSI_29-H01-M13R_c	--
SPL7	SPB-box gene, identical to squamosa promoter binding protein-like 7	AT5G18830	bCI_30-F06-M13R_c	--
CLA1	1-deoxy-D-xylulose-5-phosphate synthase	AT4G15560	bAI_15-G04-M13R_c	Transket_pyr
IPP2	IPP synthase1, isopentenyl diphosphate:dimethylallyl diphosphate isomerase	AT3G02780	bCL426Contig1	NUDIX
GGR	geranylgeranyl pyrophosphate synthase1	AT4G38460	bCL4889Contig1	--
PSY	phytoene synthase homolog1	AT5G17230	bHS_38-F04-M13R_c	SQS_PSY
PDS3	Phytoene Desaturase homolog	AT4G14210	bA_12-A10-M13R_c	--
ZDS	zeta-carotene desaturase	AT3G04870	bCL1279Contig1	--
CRTISO	carotenoid isomerase	AT1G06820	bCL1438Contig1	--
LUT2	lycopene epsilon cyclase	AT5G57030	bCL3563Contig1	Lycopene_cycl
CCD1	carotenoid cleavage dioxygenase 1	AT3G63520	bCL5417Contig1	RPE65
EIN2	ethylene-insensitive 2 (EIN2)	AT5G03280	bPS_03-F02-M13R_c	--

gene name[a]	description	Arabidopsis locus	Melogen unigene	HMMR domain
EIN3	ethylene-insensitive 3 (EIN3)	AT3G20770	bCL204Contig2	--
EIN4	Ethylene receptor, subfamily 2 (EIN4)	AT3G04580	bCL1742Contig1	Response_reg
EIN1	ethylene receptor 1 (ETR1)	AT1G66340	bCI_54-H09-M13R_c	Response_reg
ACS5	1-aminocyclopropane-1-carboxylic acid (ACC) synthase (ACS)	AT5G65800	bA_02-A09-M13R_c	--
ACS6	1-aminocyclopropane-1-carboxylic acid (ACC) synthase (ACS)	AT4G11280	bPSI_39-H08-M13R_c	--
ACS10	1-aminocyclopropane-1-carboxylate synthase, putative / ACC synthase	AT1G62960	b15d_41-D01-M13R_c	--
EAT1	1-aminocyclopropane-1-carboxylate oxidase / ACC oxidase	AT1G05010	bCL4859Contig1	2OG-FeII_Oxy
ACO1	ACO1 1-aminocyclopropane-1-carboxylate oxidase, putative / ACC oxidase	AT2G19590	bCL4923Contig1	2OG-FeII_Oxy
EXPA4	expansin, putative (EXP4) / *LeEXP1* in tomato	AT2G39700	bAI_15-F08-M13R_c	DPBB_1 Pollen_allerg_1
EXPA13	expansin, putative (EXP13)	AT3G03220	bA_39-E05-M13R_c	DPBB_1 Pollen_allerg_1
EXPL2	expansin family protein (EXPL2)	AT4G38400	bCL1936Contig1	DPBB_1 Pollen_allerg_1
EXP7	expansin, putative (EXP7)	AT1G12560	bPSI_29-G12-M13R_c	DPBB_1 Pollen_allerg_1
BGAL1	beta-galactosidase, putative / TBG4	AT3G13750	bPS_15-D10-M13R_c	--
XTR4	endo-xyloglucan transferase, putative (XTR4)	AT1G32170	bA_15-E06-M13R_c	XET_C
XTR2	endo-xyloglucan transferase (XTR2)	AT1G14720	bCL2427Contig1	Glyco_hydro_16
F18B13_25	Polygalacturonase	AT1G80170	b15d_11-F07-M13R_c	--
F28C11.9	Polygalacturonase	AT1G23460	bA_05-G07-M13R_c	--
F26K9.250	invertase/pectin methylesterase inhibitor family protein	AT3G62820	bCI_66-D03-M13R_c	PMEI
MMI9_21	invertase/pectin methylesterase inhibitor family protein	AT5G62350	bCL3362Contig1	PMEI
F26F24_4	invertase/pectin methylesterase inhibitor family protein	AT1G23205	bAI_30-D12-M13R_c	PMEI
C/VIF2	invertase/pectin methylesterase inhibitor family protein	AT5G64620	bCL1489Contig1	PMEI
MMI9_1	invertase/pectin methylesterase inhibitor family protein	AT5G62360	bCL2148Contig1	PMEI
F25I16_1	endoglucanase homolog2	AT1G18650	bCL4503Contig1	X8

gene name[a]	description	Arabidopsis locus	Melogen unigene	HMMR domain
SVP	Jointless-like MADS-box gene, short vegetative phase protein (SVP)	AT2G22540	bCL2852Contig1	SRF-TF
PI	MADS-box gene, floral homeotic protein PISTILLATA (PI)	AT5G20240	bPSI_33-F04-M13R_c	SRF-TF<br>K-box
AG	MADS-box gene, floral homeotic protein AGAMOUS (AG)	AT4G18960	bCL1793Contig1	SRF-TF<br>K-box
AGL9	SEPALLATA3-like MADS-box gene (AGL9)	AT1G24260	bCL5089Contig1	SRF-TF<br>K-box
AGL20	AGL20/SOC1-like MADS-box gene	AT2G45660	bAI_10-F02-M13R_c	SRF-TF
AGL12	AGL12-like MADS-box gene	AT1G71692	bCL2951Contig1	SRF-TF
MUF9_24	AGL62-like MADS-box gene	AT5G60440	bPSI_24-C03-M13R_c	SRF-TF
AP3	APETALA3-like MADS-box gene	AT3G54340	b46d_11-A08-M13R_c	SRF-TF<br>K-box
F13I12.140	leucine-rich repeat transmembrane protein kinase, putative, receptor kinase-like protein (Xa21)	AT3G47090	bAI_21-G07-M13R_c.	--
FLS2	Encodes a leucine-rich repeat serine/threonine protein kinase. FLS2 is involved in MAP kinase signalling relay involved in innate immunity	AT5G46330	bCI_24-G04-M13R_c	LRR_1
AtGSNOR1	Encodes a glutathione-dependent formaldehyde dehydrogenase (also known as class III type alcohol dehydrogenase)	AT5G43940	bCL3263Contig1	--
EDS1	Component of R gene-mediated disease resistance in arabidopsis with homology to eukaryotic lipases	AT3G48090	bCL3165Contig1	--
SAG101	encodes an acyl hydrolase involved in senescence	AT5G14930	bAI_26-E10-M13R_c	--
PR-1-LIKE	Encodes a PR-1-like protein. Though the Genbank record for the cDNA associated to this gene model is called 'PR-1', the sequence actually corresponds to PR-1-like	AT2G19990	bCL4983Contig1.	SCP
F17I23_340	allergen V5/Tpx-1-related family protein, similar to pathogenesis-related protein PR-1 precursor	AT4G30320	bCL820Contig1	SCP
F14M19_60	pathogenesis-related protein, putative, similar to gene PR-1 protein	AT4G25780	b15d_08-H10-M13R_c	SCP
BG1	encodes a member of glycosyl hydrolase family 17 / Beta-1,3-glucanase 1	AT3G57270	bCI_21-B07-M13R_c	Glyco_hydro_17
BG3	Beta-1,3-glucanase 3	AT3G57240	bCI_27-F09-M13R_c	Glyco_hydro_17
PR3	encodes a basic chitinase involved in ethylene/jasmonic acid mediated signalling pathway during systemic acquired resistance based on expression analyses.	AT3G12500	bAI_22-B05-M13R_c	Glyco_hydro_19
T15B16_5	chitinase, putative, similar to type II chitinase	AT4G01700	bAI_37-E08-M13R_c	Glyco_hydro_19
CTL1	chitinase-like protein 1 (CTL1), similar to class I chitinase	AT1G05850	bCL1465Contig1	Glyco_hydro_19
T9A14.6	similar to thaumatin-like protein	AT4G38660	bHS_08-A04-M13R_c	Thaumatin

gene name[a]	description	Arabidopsis locus	Melogen unigene	HMMR domain
T20H2_19	pathogenesis-related thaumatin family protein, similar to receptor serine/threonine kinase PR5K	AT1G20030	bPSI_27-C07-M13R_c	Thaumatin
CSD1	Encodes a cytosolic copper/zinc superoxide dismutase CSD1	AT1G08830	bCL306Contig2	Sod_Cu
GST6	glutathione S-transferase 6 (GST6)	AT2G47730	bCL733Contig1	GST_N<br>GST_C
GST18	Encodes glutathione transferase belonging to the zeta class of GSTs	AT2G02390	bCL2573Contig1	GST_N
GST25	Encodes glutathione transferase belonging to the tau class of GSTs. Induced by Salicylic acid	AT2G29420	bCL3039Contig1	GST_N
GST6	Encodes glutathione transferase belonging to the phi class of GSTs. Glutathione S-transferase 6 (GST6)	AT2G47730	bCL733Contig1	GST_N<br>GST_C
--	ribonuclease III family protein, similar to CAF protein (RNA helicase/RNAseIII)	AT4G15417	bPS_15-E03-M13R_c	Ribonuclease_3
DCL1	Encodes a Dicer homolog. Dicer is a RNA helicase involved in microRNA processing	AT1G01040	bPS_15-E03-M13R_c	--
AGO1	Encodes an RNA Slicer that selectively recruits microRNAs and siRNAs. There is currently no evidence that AGO1 Slicer is in a high molecular weight RNA-induced silencing complex (RISC)	AT1G48410	bCL2045Contig1	Piwi
F6F22_4	RNA-dependent RNA polymerase family protein	AT2G19930	bAI_27-E08-M13R_c	--
RDR6	Encodes RNA-dependent RNA polymerase. Involved in trans-acting siRNA and other siRNA biogenesis. Required for post-transcriptional gene silencing and natural virus resistance.	AT3G49500	bHS_19-D11-M13R_c	--
RDR2	Encodes RNA-dependent RNA polymerase that is required for endogenous siRNA (but not miRNA) formation.	AT4G11130	bCL3286Contig1	--
T31K7_7	30S ribosomal protein, putative, similar to plastid-specific 30S ribosomal protein 1, chloroplast precursor (CS-S5) (CS5) (S22) (Ribosomal protein 1) (PSRP-1)	AT5G24490	bCL4287Contig1	Ribosomal_S30AE
F24J5_17	plastid-specific 30S ribosomal protein 3, putative / PSRP-3	AT1G68590	bCL864Contig1	PSRP-3_Ycf65
EIF4E	eukaryotic translation initiation factor 4E	AT4G18040	bCL4710Contig1	IF4E
EIF(ISO)4E	eukaryotic translation initiation factor 4E, cap-binding protein	AT5G35620	bCL1086Contig1	IF4E
NCBP	novel cap-binding protein (nCBP)	AT5G18110	bCL183Contig1	IF4E
EIF4G	eukaryotic translation initiation factor 4G	AT3G60240	bCL1676Contig1	--
TOM1	encodes a host factor that is required for TMV virus multiplication.	AT4G21790	bPS_10-H03-M13R_c	DUF1084
TOM2A	TOM2A encodes a 280 amino acid putative four-pass transmembrane protein with a C-terminal farnesylation signal, essential for efficient multiplication of tobacco mosaic viruses.	AT1G32400	bCL3115Contig1	--
TOM 3	tobamovirus multiplication protein 3, putative TOM3, putative THH1	AT1G14530	bPSI_36-E03-M13R_c	DUF1084

[a]Gene name according to nomenclature proposed in The Arabidopsis Information Resource (TAIR) [6].
